# Is This Car Looking at You? How Anthropomorphism Predicts Fusiform Face Area Activation when Seeing Cars

**DOI:** 10.1371/journal.pone.0113885

**Published:** 2014-12-17

**Authors:** Simone Kühn, Timothy R. Brick, Barbara C. N. Müller, Jürgen Gallinat

**Affiliations:** 1 Center for Lifespan Psychology, Max Planck Institute for Human Development, Lentzeallee 94, 14195, Berlin, Germany; 2 Behavioural Science Institute, Radboud University of Nijmegen, P. O. Box 9104, 6500 HE, Nijmegen, Netherlands; 3 Department of Psychology, Ludwig-Maximilian University, Leopoldstrasse 13, 80802, München, Germany; 4 Clinic for Psychiatry and Psychotherapy, Charité University Medicine, St. Hedwig-Krankenhaus, Große Hamburger Straße 5–11, 10115, Berlin, Germany; 5 Clinic and Policlinic for Psychiatry and Psychotherapy, University Clinic Hamburg-Eppendorf, Martinistraße 52, 20246, Hamburg, Germany; University of Vienna, Austria

## Abstract

Anthropomorphism encompasses the attribution of human characteristics to non-living objects. In particular the human tendency to see faces in cars has long been noticed, yet its neural correlates are unknown. We set out to investigate whether the fusiform face area (FFA) is associated with seeing human features in car fronts, or whether, the higher-level theory of mind network (ToM), namely temporoparietal junction (TPJ) and medial prefrontal cortex (MPFC) show a link to anthropomorphism. Twenty participants underwent fMRI scanning during a passive car-front viewing task. We extracted brain activity from FFA, TPJ and MPFC. After the fMRI session participants were asked to spontaneously list adjectives that characterize each car front. Five raters judged the degree to which each adjective can be applied as a characteristic of human beings. By means of linear mixed models we found that the implicit tendency to anthropomorphize individual car fronts predicts FFA, but not TPJ or MPFC activity. The results point to an important role of FFA in the phenomenon of ascribing human attributes to non-living objects. Interestingly, brain regions that have been associated with thinking about beliefs and mental states of others (TPJ, MPFC) do not seem to be related to anthropomorphism of car fronts.

## Introduction

In daily life it is fairly common that people see human elements in non-human objects: we see faces in clouds, give names to our cars, or scold malfunctioning computers. This so-called tendency to anthropomorphize pervades human judgement [1]. Although the tendency to anthropomorphize is pervasive, people do not anthropomorphize all objects spontaneously, nor are they able to anthropomorphize different objects with equal ease. The literature suggests that the ability to anthropomorphize may depend on the presence of specific features (e.g., Epley, Waytz, & Cacioppo, 2007), with an increase in human features leading to an increase in anthropomorphism. The car features that make people ascribe certain human traits to car fronts, such as maturity, sex, and interpersonal attitudes are similar to those found with human faces [2] and similar across different cultures [3]. Previous research has shown that eye movements while watching car fronts resemble those when seeing faces [4]. The number of fixations was found to be greatest on the cars headlights and the eyes of the face; even when participants were asked to make judgements about other regions of the car and the face. A predominance of fixation on the eyes in face perception is known within the existing literature. The fact that the same phenomenon occurs in car perception has been interpreted as evidence in favour of the existence of an over-perception error, in which cars are processed similar to faces (Windhager et al., 2010). However, the face-like fixation pattern has not been directly associated with a subjective rating of humanness, evidence that would be crucial to establish the link to the perception of human features in non-living objects. It may therefore well be that the eye movement pattern reflect visual features of car fronts, not necessarily anthropomorphism.

Within the scope of the present study we set out to investigate the neural basis of anthropomorphism. In the neuroscientific literature activity in the so-called fusiform face area (FFA) measured by means of blood-oxygen level dependent (BOLD) response in functional magnetic resonance imaging (fMRI) has been associated with face processing. Multiple neuroimaging studies have reported the fusiform gyrus to be more active during face rather than object viewing [5]–[7]. Therefore we presume that FFA could be related to anthropomorphism.

In contrast to FFA, one may reason that the higher-level brain areas known to be responsible for the attribution of beliefs to others could represent another domain of anthropomorphism. Neuroimaging research targeting so-called theory of mind (ToM) reasoning has provided extensive evidence suggesting that a consistent set of brain regions is recruited when participants are required to reason about other people. This brain network (also termed the “social brain network”) includes the medial prefrontal cortex (MPFC), the bilateral temporo-parietal junction (TPJ), the superior temporal sulcus and the temporal poles [8]–[11]. In particular, two brain areas within the social brain network have been claimed to be crucial for ToM, namely the TPJ and MPFC. These brain areas are assumed to have well defined roles in reasoning about other people's mental states. Specifically, Frith and Frith [8] have argued that the MPFC is involved in decoupling mental states from physical state representations and in particular the right TPJ is thought to be involved in reasoning about other people's representational mental states [12]. Additionally, a recent study has demonstrated a link between inter-individual differences in self-reported anthropomorphism and grey matter volume in the left TPJ that is part of the so-called ToM network [13].

Our aim of the present study was to investigate whether the neural basis of anthropomorphism in cars is located in perceptual brain areas or regions related to higher order processing of mental states. Therefore we set out to address the question of whether brain areas involved in face perception or ToM processing are activated when seeing cars depending on the degree of anthropomorphism of the specific car seen.

## Methods

### Participants

Twenty healthy young adults (age: mean  = 25.55 years, ranging from 19 to 33, 10 females) who reported no special interest in cars participated after having given written informed consent. The study was conducted according to the Declaration of Helsinki, with approval of the German Psychological Society ethics committee. All participants had normal or corrected-to-normal vision, and no history of neurological, major medical, or psychiatric disorder. All participants were right-handed.

### Scanning Procedure

Images were collected on a 3T Magnetom Trio MRI scanner system (Siemens Medical Systems, Erlangen, Germany) using a 32-channel radiofrequency head coil. The structural images were obtained using a three-dimensional T1-weighted magnetization prepared gradient-echo sequence (MPRAGE) based on the ADNI protocol (www.adni-info.org) (repetition time (TR)  = 2500 ms; echo time (TE)  = 4.77 ms; TI  = 1100 ms, acquisition matrix  = 256×256×176, flip angle  = 7°; 1×1×1 mm voxel size). Functional images were collected using a T2*-weighted echo planar imaging (EPI) sequence sensitive to blood oxygen level dependent (BOLD) contrast (TR  = 2000 ms, TE  = 30 ms, image matrix  = 64×64, FOV  = 216 mm, flip angle  = 80°, voxel size 3×3×3 mm^3^, 36 axial slices).

### Materials and Tasks

**Figure 1:**
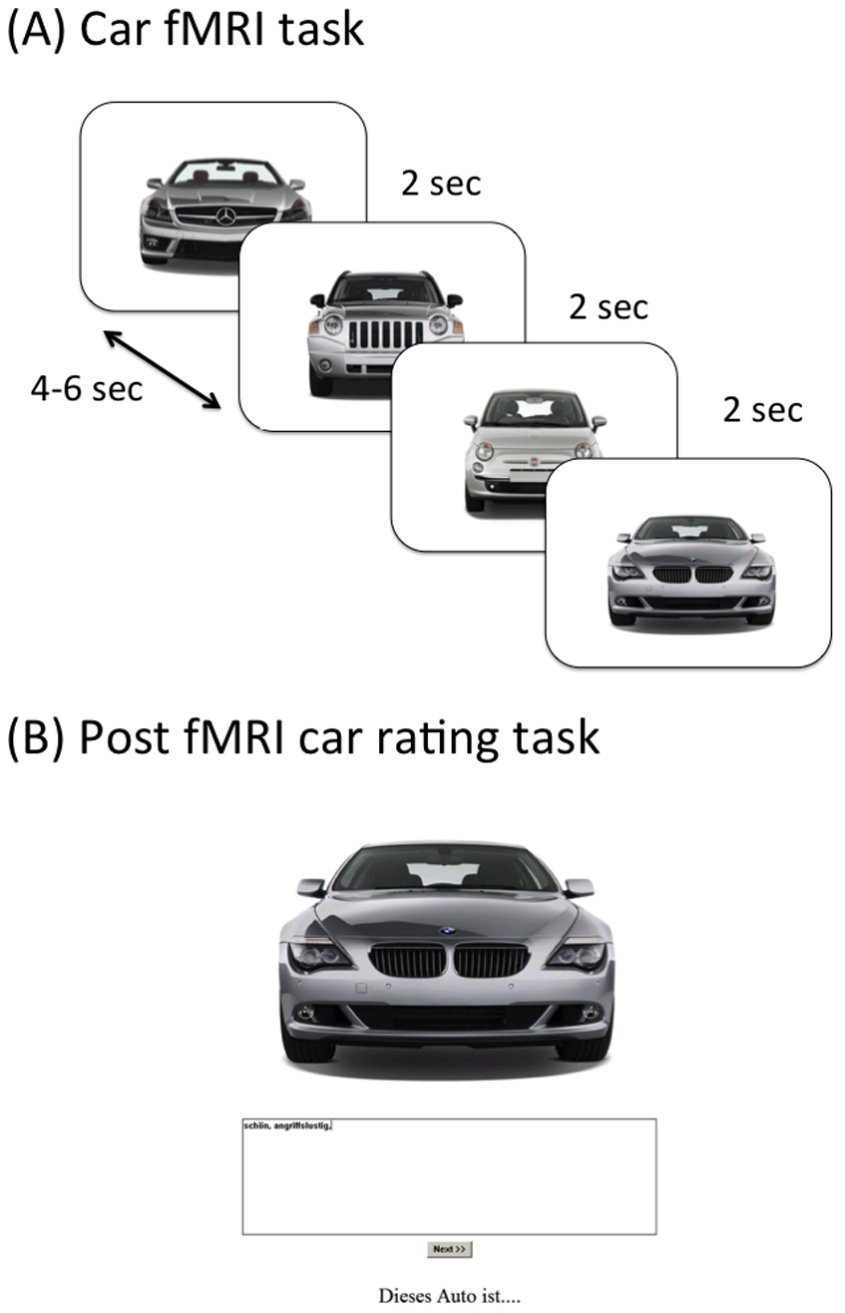
Car stimuli shown during fMRI scanning (A). Post-scanner rating of cars, where participants entered adjectives that characterize the car (“This car is …”) (B).

Each participant underwent two tasks in the scanner, a car task and a face/house localizer, with identical procedures but different picture stimuli. Each type of stimulus (cars or faces/houses) was presented in a separate run. For the car task, we selected 50 pictures of cars (color images of grey cars) depicting the front of 3D models (for an example see *Fig. 1A*). For the face-house localizer we used 26 pictures of houses and 26 pictures of faces from the Radboud face database (female and male) [14]. Each trial started with a presentation of one of the pictures for 2 seconds. After a jitter interval between 4 to 6 seconds (varied in steps of 500 ms) the next object was presented.

Outside of the scanner all cars were presented again. Borrowing from Epley's procedure to assess anthropomorphism [15], we used an implicit measure where participants were prompted to enter adjectives that describe the car best (“This car is …”) (*Fig. 1B*). The number of adjectives was not specifically restricted. However, the range was narrow and participants named between 1 and 6 adjectives. The generated adjectives were rated on the basis of their applicability to humans by five raters, who rated independently from one another on a scale from 0 =  no adjectives that could be applied to characterize human beings, to 6 =  all adjectives characterize human beings. E.g. adjectives like “feminine”, “elegant” and “childish” were rated as applicable to humans, whereas adjectives such as “expensive”, “space-saving” and “rural” were rated as not applicable to humans. The mean rating across all raters was computed as the so-called implicit anthropomorphism score per car and per participant. Since the anthropomorphism scores showed a considerable amount of variability for individual cars (average SD  = 1.48, average range  = 4.94 across all cars) across different subjects, we decided to account for idiosyncratic anthropomorphism instead of averaging across subjects to obtain a single score per car.

### fMRI Data Pre-processing and Main Analysis

The fMRI data were analysed using SPM8 software (Wellcome Department of Cognitive Neurology, London, UK). The first four volumes of all EPI series were excluded from the analysis to allow the magnetization to approach a dynamic equilibrium. Data processing started with slice time correction and realignment of the EPI datasets. A mean image for all EPI volumes was created, to which individual volumes were spatially realigned by means of rigid body transformations. The structural image was co-registered with the mean image of the EPI series. Then the structural image was normalized to the Montreal Neurological Institute (MNI) template, and the normalization parameters were applied to the EPI images to ensure an anatomically informed normalization. A commonly applied filter of 8 mm FWHM (full-width at half maximum) was used. Low-frequency drifts in the time domain were removed by modelling the time series for each voxel by a set of discrete cosine functions to which a cut-off of 128 seconds was applied. We employed a general linear model (GLM) in which we modelled the visually presented objects separately. After normalization, these vectors were convolved with a canonical hemodynamic response function (HRF) and its temporal derivatives to form the design matrix. The parameters of the ensuing general linear model were estimated in the usual way and used to form contrasts between faces and houses to derive FFA. The resulting contrast images were then entered into a series of one sample T-tests at the second (between-subject) level. For the parametric analysis the subject specific anthropomorphism scores for each car were entered as a parameter in the first-level analysis.

For display purposes the resulting statistical parametric maps (SPMs) were thresholded at *p*<0.001 (cluster size >10). The resulting maps were overlaid onto a normalized T1 weighted MNI template (colin27) and the coordinates reported correspond to the MNI coordinate system.

### Meta-Analysis ToM brain regions

Since there is no agreed-upon localizer for the ToM network we conducted an activation-likelihood estimation meta-analysis (ALE, [16] on 26 studies on mentalizing that reported 31 peaks of activation in the proximity of TPJ and 31 in medial prefrontal cortex (MPFC) (see [11] for the references). We used a threshold with a false-discovery rate (FDR) of *p*<0.01 and a cluster size above 200 mm^3^. The cluster identified in TPJ was centred around the coordinate (56, −47, 33) a (cluster size: 4448 mm^3^) and we used the mirrored ROI for the localization of left TPJ. The literature-based MPFC ROI was located at (2, 53, 13) (cluster size: 3368 mm^3^).

### ROI extraction

We used the data from faces and houses as a localizer task in order to determine FFA. On a group level we contrasted BOLD activity in response to faces compared to houses, thresholded the contrast at *p*<0.001 (uncorrected) and extracted FFA on the left (−42, −46, −20) and right (42, −46, −17) hemisphere. For left and right TPJ as well as MPFC we used the meta-analysis based ROIs described above. From these ROIs we extracted mean percent signal change over a time window of 4–6 seconds after each car stimulus onset for each subject (http://marsbar.sourceforge.net/, [17].

### Linear mixed effects analysis

To analyse the relationship between brain activity in the predefined ROIs and the anthropomorphism score we used mixed-effects regression using lme4 [18] in R with random intercepts for subject and car. This predicts the brain activity for each individual's viewing of each car from that individual's anthropomorphism score for that car, controlling for both the individual's mean brain activity across all cars and for that car's effect on brain activity across all individuals. Conceptually, this allows us to examine the unique influences on brain activity corresponding to person-specific differences in ratings of anthropomorphism.

This means as fixed effects, we entered the implicit anthropomorphism for each car into the model. As random effects, we had intercepts for subjects and cars (brain activity ∼ anthropomorphism + (1|subject) + (1|car)). Visual inspection of residual plots did not reveal any obvious deviations from homoscedasticity or normality. We assessed whether the models were fitting to the data by using likelihood ratio tests, calculated as −2(l_0_–l_1_) where l_0_ and l_1_ denote the maximized log-likelihood of two models to be compared (called I_0_ and I_1_, respectively). The two models are chosen so that I_1_ includes the predictor of interest (anthropomorphism score); I_0_ differs from I_1_ only by removing the influence of this predictor. This statistic has a null distribution approximating that of **χ^2^**, with degrees of freedom obtained from the difference in the number of parameters. A **χ^2^** test can therefore assess whether a predictor contributes significantly to the model's fit, with a significant result justifying the addition of the predictor to the model. This approach allows us to investigate whether the prediction of the activity in a certain brain region of interest can be significantly improved by adding the degree of anthropomorphism of the car.

### Geometric Morphometrics

In order to explore and visualize the relationship between anthropomorphism score and car shape we defined 34 landmarks [2]. The sets of landmarks, were digitized using the *R* geomorph toolbox and were superimposed using Generalized Procrustes analysis (GPA, [19]. The resulting shape coordinates were then regressed onto the anthropomorphism score across participants using partial least squares (PLS) regression [20], and outlines of predicted prototypical high- and low-anthropomorphism cars were generated.

## Results

**Figure 2:**
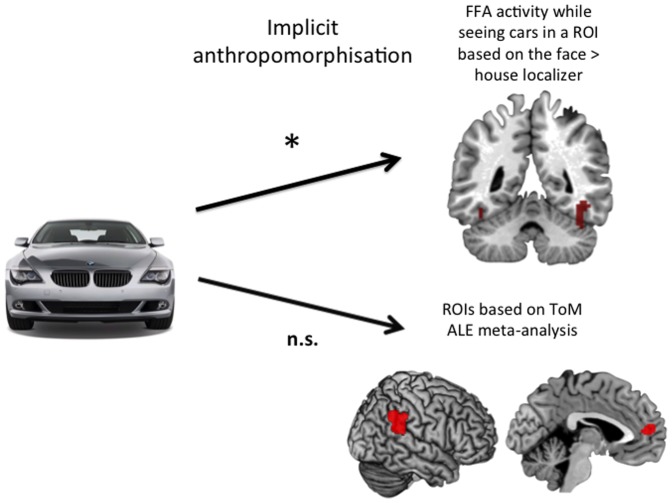
Schematic illustration of the results of the linear mixed model, where prediction of bilateral fusiform face area (FFA) activation benefits from the inclusion of the individual's degree of anthropomorphism of the specific car, whereas the prediction of temporoparietal junction (TPJ) and medial prefrontal cortex (MPFC) does not seem to be associated to the tendency to anthropomorphize.

The inter-rater reliability for the implicit anthropomorphism score derived from the adjectives that participants generated in face of each car was remarkably high (Chronbach's alpha  = 0.88). We set up separate linear mixed models to predict brain activity in bilateral FFA and in brain regions of the ToM network: right and left TPJ and MPFC. The model predicting bilateral FFA activity during car viewing from the implicit anthropomorphism score was significantly better fit than the one without this predictor (**χ^2^**(1)  = 16.65, *p*<0.001; contribution of the predictor according to Satterthwaite approximation for degrees of freedom [21]: *t*(855.8)  = 4.10, *p*<0.001). The prediction of the activity in ToM regions did not improve when adding the implicit anthropomorphism score as a predictor (left TPJ: **χ^2^**(1)  = 1.18, *p* = 0.278; contribution of the predictor *t*(817.1)  = 1.09, *p* = 0.277; right TPJ: **χ^2^**(1)  = 0.35, *p* = 0.557; contribution of the predictor *t*(908.6)  = 0.59, *p* = 0.557; MPFC: **χ^2^**(1)  = 1.57, *p* = 0.210; contribution of the predictor *t*(726.3)  = 1.26, *p* = 0.207). To summarize, the results indicate that anthropomorphism does contribute significantly to the prediction of brain activity in bilateral FFA during car processing, but not in the ToM networks (*Figs. 2* *and* *3*).

**Figure 3:**
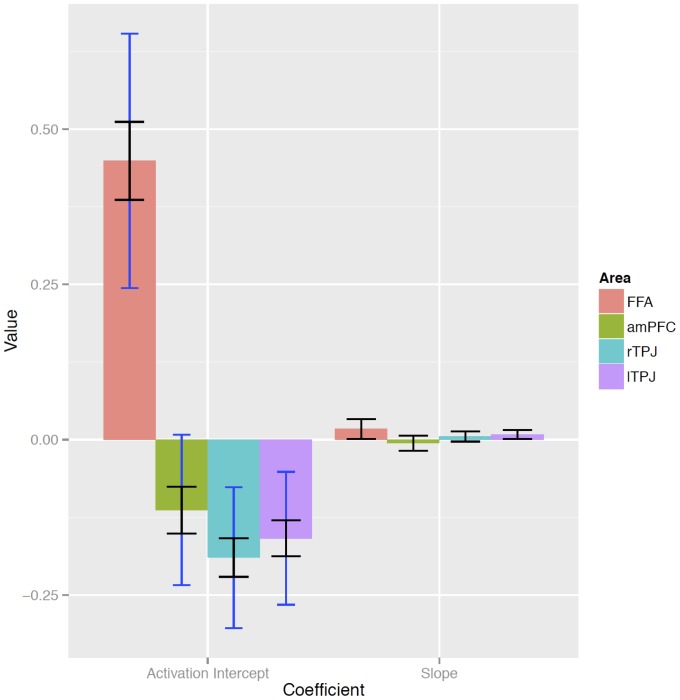
Plot of brain activation intercept and slope in a random-intercept model predicting activation from car anthropomorphism values. Black error bars indicate standard errors of the fixed effects, blue bars indicate standard deviations of the random intercepts.

**Figure 4:**
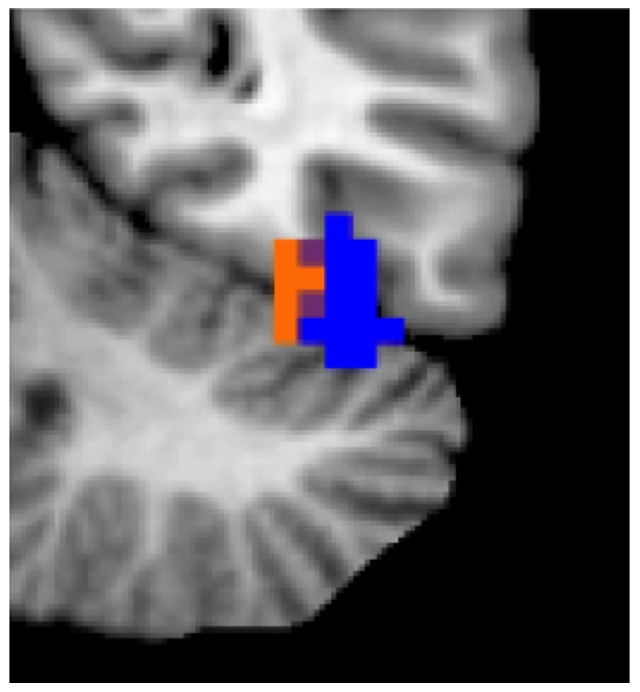
Right FFA activation resulting from a whole brain parametric analysis with the anthropomorphism score for each car (in orange) and the activity resulting from the independent localizer for right FFA based on the contrast faces vs. houses (in blue) is depicted.

To confirm the abovementioned theory-driven ROI analyses and to test whether any additional brain regions are associated with implicit anthropomorphisation we ran a whole brain parametric analysis using the car and subject specific anthropomorphism scores as a parameter. A positive association between the anthropomorphism regressor and BOLD activity was observed in right FFA (36 −52 −23, *p*<0.001, cluster >10). This cluster in right FFA is partly overlapping with the right FFA ROI derived from the localizer contrast faces vs. houses (*Fig. 4*). No other brain region reached significance, neither in this, nor in the reverse contrast.

**Figure 5:**
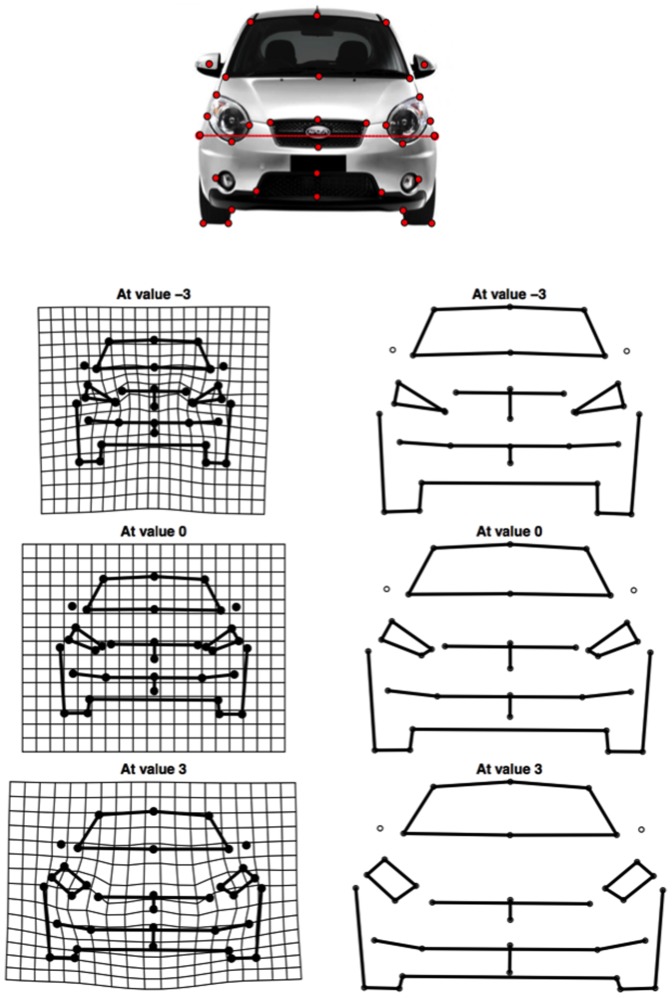
Shape-anthropomorphism PLS analysis visualizing the mean and ±3 SDs along the first pair of PLS axes.

To visualize car fronts that result in high or low anthropomorphism scores we applied a PLS analysis to the correlation matrix between the shape of each car (as represented by procrustes-aligned landmark locations) and the average anthropomorphism score for that car. A permutation test was performed using the PLS Matlab toolbox (Krishnan et al., 2011) and found one significant latent variable (*p*<0.05). Following these findings, we performed PLS regression using the R PLS package (Bjørn-Helge, 2013) to predict point scores from anthropomorphism values using a single latent variable. The latent variable explained 20.08% of variation in shape point locations and 33.34% percent of variation in anthropomorphism scores. We then used the fitted model to predict point locations for a car with anthropomorphism at mean, or three standard deviations above or below the mean, producing a caricature of car shapes that would show a prototypical mean, high-anthropomorphism and low-anthropomorphism response. *Fig. 5* shows the predicted shapes, both in outline form and using a thin-plate spline fit to better illustrate the differences in shape. The high-anthropomorphism cars are predicted to have a wider stance, a lower position of the grille, and round or square rather than triangular headlights.

## Discussion

Our results demonstrate an association between neural activation in bilateral FFA, the face-sensitive brain region within the fusiform gyrus, while viewing car fronts and the tendency of participants to characterize the same car fronts with adjectives that apply to humans. This association was confirmed by means of a whole brain parametric analysis showing a positive relationship between anthropomorphism scores and brain activity in right FFA. In contrast, the higher-level brain regions that constitute the ToM network and have been associated with mentalizing, namely left and right TPJ and the MPFC do not show this link to anthropomorphism. For illustration purposes geographic morphometrics were used to compute prototypical cars that elicit high compared to low levels of anthropomorphism scores.

The result of the present study suggests that FFA activity constitutes the neural basis of anthropomorphism of car fronts, implying that the attribution of human traits and features relies on brain regions localized comparably early in the visual processing stream. Particularly interesting is the fact that this is the case although the anthropomorphism scores show considerable variability within particular cars across different subjects. We therefore conclude that it is in particular the idiosyncratic anthropomorphic perception of the physiological properties of the cars that drive FFA activity. At the same time higher cognitive brain regions of the ToM network, that have been shown to activate when people think about human beings and attribute mental states to others [8], [11] do not seem to play a prominent role in anthropomorphism of car fronts. This may be seen as in line with the previous study showing similar eye movement patterns when viewing car fronts and human faces [4]. The authors explain their results by suggesting that the evolved patterns of face detection and attention orientation towards facial expressions may lead to an over-perception error that triggers the same mechanisms when processing car fronts. Potentially this mechanism also applies to the activity in FFA, which has been suggested as an inborn module for face perception.

However, the selectivity with which FFA is activated by faces only has been called into question recently. Some researchers argue that FFA discriminates between any familiar stimuli. Several lines of research converge to suggest that level of categorization and expertise account for a large part of the activation difference between faces and objects. It has been shown that non-face objects elicit more activation in the FFA when matched to specific labels as compared to more categorical ones (e.g. “ketchup bottle” vs. “bottle”, [22]. In line with this finding, experts in animal-like objects, such as birds or cars show strong activity in FFA [23]–[25]. Moreover, based on studies on autism spectrum disorder it has been speculated that the expertise framework of face processing is better suited to explain why autistic persons fail to develop cortical face specialization due to their reduced social interest in other human beings [26]. Because of the debate that FFA is activated by familiar, not necessarily face-like stimuli, we recruited participants with no particular interest in cars to avoid influences due to the previously reported effects of expertise. Furthermore we focussed on within-subject variations between FFA activation elicited by different car fronts, not on between-subject differences that may indeed be affected by different levels of expertise. The fact that we do find an association between the degree to which participants use human-like attributes to characterize the car and FFA activity while viewing car fronts can be interpreted in favour of the classical involvement of FFA in face specific processing. However, the attributes that participants listed were mostly not characteristic of faces, but applicable to humans in general; therefore one may argue that the fact that particular car fronts lead to the recall of adjectives that characterize humans may be an instance of objects being associated with more specific labels than usual cars. That may in comparison mainly elicit superficial adjectives such as “black” or “shiny” when the car body appears in black colour or well polished. This higher level of expertise or the higher degree of holistic processing [27], [28] that may be elicited when human attributes are recalled may be the cause of the stronger FFA activity, not the face-ness of the car front itself.

From the present data we can conclude that a brain area that computes comparably lower level information, not the higher-level mentalizing network consisting of MPFC and TPJ activate in association with implicit anthropomorphism. This stands in contrast to the structural grey matter findings of Cullen and colleagues [13], who report a positive association between inter-individual differences in a self-report anthropomorphism questionnaire (IADQ, [29]) and grey matter in left TPJ. However two obvious differences protrude: First of all the studies differ by means of the dependent variable; while we focus on brain activation Cullen and colleagues investigated brain structure. The complex interplay between brain structure and function is still not unequivocally resolved. Secondly, we assessed anthropomorphism by means of an implicit procedure. That is, the participants did not know what our research focus was at the point when they listed adjectives characteristic of the car fronts shown. In the structural imaging study on the other hand, a questionnaire was used that does not hide the target of assessment and might therefore lead to different kinds of response biases. Third, our present study relied on within-subject variations of activity, whereas the analysis presented by Cullen targeted inter-individual differences.

Future research is needed to investigate the difference in the neural correlates of implicit compared to explicit measures of the tendency to anthropomorphize and the morphometrics that these differences are based on. Moreover, the research should be extended to non-car objects in order to explore whether the association between FFA activity and instances of anthropomorphism also exists across a broader range of object categories. To explore the car features that elicit high anthropomorphism scores in more detail, one could extract these eliciting features from the car morphologies and present prototypical cars rebuilt according to these rules in a parametric fMRI design that should elicit predictable degrees of FFA activation.

## References

[1] Guthrie S (1993) Faces in the Clouds. Oxford University Press.

[2] Windhager S, Slice DE, Schaefer K, Oberzaucher E, Thorstensen T, et al (2008) Face to Face. Hum Nat 19:331–346 10.1007/s12110-008-9047-z 26181746

[3] Windhager S, Bookstein FL, Grammer K, Oberzaucher E, Said H, et al (2012) “Cars have their own faces”: cross-cultural ratings of car shapes in biological (stereotypical) terms. Evolution and Human Behavior 33:109–120 10.1016/j.evolhumbehav.2011.06.003

[4] Windhager S, Hutzler F, Carbon C-C, Oberzaucher E, Schaefer K, et al (2010) Laying Eyes on Headlights: Eye Movements Suggest Facial Features in Cars. Collegium Antropologicum 34:1075–1080. 20977106

[5] Haxby JV, Horwitz B, Ungerleider LG (1994) The functional organization of human extrastriate cortex: a PET-rCBF study of selective attention to faces and locations. Journal of Neuroscience: 6336–6353. 10.1523/JNEUROSCI.14-11-06336.1994 PMC6577268 7965040

[6] Kanwisher N, McDermott J, Chun MM (1997) The fusiform face area: a module in human extrastriate cortex specialized for face perception. Journal of Neuroscience: 4302–4311. 10.1523/JNEUROSCI.17-11-04302.1997 PMC6573547 9151747

[7] Kanwisher N, Yovel G (2006) The fusiform face area: a cortical region specialized for the perception of faces. Philosophical Transactions of the Royal Society B: Biological Sciences 361:2109–2128 10.1016/j.cub.2005.04.031 PMC1857737 17118927

[8] Frith U (2003) Development and neurophysiology of mentalizing. Philosophical Transactions of the Royal Society B: Biological Sciences 358:459–473 10.1098/rstb.2002.1218 PMC1693139 12689373

[9] Saxe R, Kanwisher N (2003) People thinking about thinking peopleThe role of the temporo-parietal junction in “theory of mind”. NeuroImage 19:1835–1842 10.1016/S1053-8119(03)00230-1 12948738

[10] Ruby P, Decety J (2003) What you believe versus what you think they believe: a neuroimaging study of conceptual perspective-taking. European Journal of Neuroscience 17:2475–2480 10.1046/j.1460-9568.2003.02673.x 12814380

[11] Brass M, Ruby P, Spengler S (2009) Inhibition of imitative behaviour and social cognition. Philosophical Transactions of the Royal Society B: Biological Sciences 364:2359–2367 10.1016/S0149-7634(01)00014-8 PMC2865080 19620107

[12] Saxe R (2006) Uniquely human social cognition. Current Opinion in Neurobiology 16:235–239 10.1016/j.conb.2006.03.001 16546372

[13] Cullen H, Kanai R, Bahrami B, Rees G (2013) Individual differences in anthropomorphic attributions and human brain structure. Social Cognitive and Affective Neuroscience. doi:10.1093/scan/nst109. PMC4158361 23887807

[14] Langner O, Dotsch R, Bijlstra G, Wigboldus DHJ, Hawk ST, et al (2010) Presentation and validation of the Radboud Faces Database. Cognition & Emotion 24:1377–1388 10.1080/02699930903485076

[15] Epley N, Akalis S, Waytz A, Cacioppo JT (2008) Creating Social Connection Through Inferential Reproduction: Loneliness and Perceived Agency in Gadgets, Gods, and Greyhounds. Psychological Science 19:114–120 10.1111/j.1467-9280.2008.02056.x 18271858

[16] Eickhoff SB, Laird AR, Grefkes C, Wang LE, Zilles K, et al (2009) Coordinate-based activation likelihood estimation meta-analysis of neuroimaging data: A random-effects approach based on empirical estimates of spatial uncertainty. Human Brain Mapping 30:2907–2926 10.1002/hbm.20718 19172646 PMC2872071

[17] Brett M, Anton J-C, Valabreuge R, Poline JB (2002) Region of interest analysis using an SPM toolbox. Presented at the 8th International Conference on Functional Mapping of the Human Brain.

[18] Bates D, Maechler M, Ben Bolker (2012) lme4: Linear mixed-effects models using S4 classes. (2012).

[19] Gower JC (1975) Generalized procrustes analysis. Psychometrika 40:33–51 10.1007/BF02291478

[20] Corti FJ, Rohlf M (2000) Use of Two-Block Partial Least-Squares to Study Covariation in Shape. Systematic Biology 49:740–753 10.1080/106351500750049806 12116437

[21] Satterthwaite FE (1946) An approximate distribution of estimates of variance components. Biometrics bulletin. 20287815

[22] Gauthier I, Anderson AW, Tarr MJ, Skudlarski P, Gore JC (1997) Levels of categorization in visual recognition studied using functional magnetic resonance imaging. Current Biology 7:645–651 10.1016/S0960-9822(06)00291-0 9285718

[23] Gauthier I, Tarr MJ, Anderson AW, Skudlarski P, Gore JC (1999) Activation of the middle fusiform “face area” increases with expertise in recognizing novel objects. Nature Neuroscience 2:568–573 10.1038/9224 10448223

[24] Gauthier I, Skudlarski P, Gore JC, Anderson AW (2000) Expertise for cars and birds recruits brain areas involved in face recognition. Nature Neuroscience 3:191–197 10.1038/72140 10649576

[25] McGugin RW, Gatenby JC, Gore JC, Gauthier I (2012) High-resolution imaging of expertise reveals reliable object selectivity in the fusiform face area related to perceptual performance. 10.1073/pnas.1116333109 PMC3479484 23027970

[26] Grelotti DJ, Gauthier I, Schultz RT (2002) Social interest and the development of cortical face specialization: What autism teaches us about face processing. Dev Psychobiol 40:213–225 10.1002/dev.10028 11891634

[27] Farah MJ, Wilson KD, Drain M, Tanaka JN (1998) What is“ special” about face perception? Psychological Review. 10.1037/0033-295x.105.3.482 9697428

[28] Richler JJ, Cheung OS, Gauthier I (2011) Holistic Processing Predicts Face Recognition. Psychological Science 22:464–471 10.1177/0956797611401753 21393576 PMC3077885

[29] Waytz A, Cacioppo J, Epley N (2010) Who Sees Human?: The Stability and Importance of Individual Differences in Anthropomorphism. Perspectives on Psychological Science 5:219–232 10.1177/1745691610369336 24839457 PMC4021380

